# Recurrence and Survival After Minimally Invasive and Open Esophagectomy for Esophageal Cancer: A Post Hoc Analysis of the Ensure Study

**DOI:** 10.1097/SLA.0000000000006280

**Published:** 2024-04-05

**Authors:** Sofie P.G. Henckens, Nannet Schuring, Jessie A. Elliott, Asif Johar, Sheraz R. Markar, Amaia Gantxegi, Pernilla Lagergren, George B. Hanna, Manuel Pera, John V. Reynolds, Mark I. van Berge Henegouwen, Suzanne S. Gisbertz, on behalf of the ENSURE study group

**Affiliations:** * Department of Surgery, Amsterdam UMC, University of Amsterdam, Meibergdreef, Amsterdam, the Netherlands; † Cancer Center Amsterdam, Department of Cancer Treatment and Quality of Life, Amsterdam, the Netherlands; ‡ Department of Gastroenterology and Hepatology, Amsterdam UMC, University of Amsterdam, Amsterdam Gastroenterology Endocrinology Metabolism, Meibergdreef, Amsterdam, the Netherlands; § Department of Surgery, Trinity Centre for Health Sciences, St. James's Hospital and Trinity College Dublin, Dublin, Ireland; ∥ Department of Molecular Medicine and Surgery, Karolinska Institutet, Karolinska University Hospital, Stockholm, Sweden; ¶ Nuffield Department of Surgical Sciences, Surgical Intervention Trials Unit, University of Oxford, Oxford, UK; # Department of Surgery, Vall d’Hebron University Hospital, Universitat Autònoma de Barcelona, Barcelona, Spain; ** Department of Surgery, Imperial College London, London, UK; †† Department of Surgery, Section of Gastrointestinal Surgery, Hospital del Mar, Universitat Autònoma de Barcelona, Hospital del Mar Medical Research Institute (IMIM), Barcelona, Spain

**Keywords:** esophageal cancer, esophagectomy, minimally invasive surgery, recurrence, survival

## Abstract

**Objective:**

To determine the impact of operative approach [open (OE), hybrid minimally invasive (HMIE), and total minimally invasive (TMIE) esophagectomy] on operative and oncologic outcomes for patients treated with curative intent for esophageal and junctional cancer.

**Background:**

The optimum oncologic surgical approach to esophageal and junctional cancer is unclear.

**Methods:**

This secondary analysis of the European multicenter ENSURE study includes patients undergoing curative-intent esophagectomy for cancer between 2009 and 2015 across 20 high-volume centers. Primary endpoints were disease-free survival (DFS) and the incidence and location of disease recurrence. Secondary endpoints included among others R0 resection rate, lymph node yield, and overall survival (OS).

**Results:**

In total, 3199 patients were included. Of these, 55% underwent OE, 17% HMIE, and 29% TMIE. DFS was independently increased post-TMIE [hazard ratio (HR): 0.86 (95% CI: 0.76–0.98), *P* = 0.022] compared with OE. Multivariable regression demonstrated no difference in absolute locoregional recurrence risk according to the operative approach [HMIE vs OE, odds ratio (OR): 0.79, *P* = 0.257; TMIE vs OE, OR: 0.84, *P* = 0.243]. The probability of systemic recurrence was independently increased post-HMIE (OR: 2.07, *P* = 0.031), but not TMIE (OR: 0.86, *P* = 0.508). R0 resection rates (*P* = 0.005) and nodal yield (*P* < 0.001) were independently increased after TMIE, but not HMIE (*P* = 0.424; *P* = 0.512) compared with OE. OS was independently improved following both HMIE (HR: 0.79, *P* = 0.009) and TMIE (HR: 0.82, *P* = 0.003) as compared with OE.

**Conclusion:**

In this European multicenter study, TMIE was associated with improved surgical quality and DFS, whereas both TMIE and HMIE were associated with improved OS as compared with OE for esophageal cancer.

Despite optimization of perioperative strategies, increasing specialization, and centralization of care, esophagectomy remains an exemplar of a complex surgical intervention, associated with significant risks of major perioperative morbidity and mortality. The most recent report from Esodata, including over 6000 patients across 39 international centers, and utilizing the definitions described by the Esophagectomy Complications Consensus Group, highlights a 61% complication rate, and 30 and 90-day mortality of 2% and 4.5%, respectively.^1^

The drive to improve perioperative care for patients with esophageal cancer has led to the development of enhanced recovery pathways, facilitated in the modern era by the advancement of minimally invasive techniques for esophagectomy with lymphadenectomy including hybrid minimally invasive esophagectomy (HMIE) and total minimally invasive esophagectomy (TMIE) approaches.^2^ Several beneficial short-term outcomes have been demonstrated after minimally invasive esophagectomy, compared with open esophagectomy (OE).^3,4^ Minimally invasive approaches have been shown to result in decreased postoperative pulmonary morbidity and faster recovery as compared with after OE, as described in the multicenter, open-label, randomized controlled traditionally invasive vs. minimally invasive esophagectomy and multicentre randomized controlled phase III trial trials.^5–7^ After the publication of these trials, minimally invasive approaches have been adopted in several international high-volume centers.^5,8^ However, implementation has been limited by concerns regarding the generalizability of trial findings because they could not be confirmed in an external validation study after the nationwide implementation of minimally invasive techniques in the Netherlands.^9^ Minimal invasive surgery is associated with a significant learning curve, and several studies have indicated a higher risk of anastomotic leakage and reoperation after MIE compared with after OE.^10–14^ As such, there remains significant global heterogeneity in operative approach, with 52% of cases in the recent Esodata report being performed with an OE, 23% HMIE, and 25% TMIE.^1^ Further large-scale studies are needed to determine the impact of increasing international implementation of minimally invasive approaches on short-term outcomes after esophagectomy.

Furthermore, despite recent advances, after completion of curative-intent treatment for esophageal cancer, approximately half of patients will develop recurrent disease.^7,15,16^ Knowledge of recurrence patterns after the previously mentioned 3 surgical approaches of esophagectomy could provide useful information on the effectiveness of treatment. Several reports now indicate that minimally invasive approaches may be associated with improved nodal yields, and in particular thoracic lymph node dissection, with some studies showing improvements in R0 resection rates after MIE as compared with OE.^17,18^ However, little is known as to whether differences in surgical quality indicators translate into differences in recurrence patterns, distant treatment failure, and ultimately survival. This international multicenter collaborative study conducted across 20 European and North American high-volume centers aimed to determine the impact of operative approach (OE, HMIE, or TMIE) on operative and oncologic outcomes for patients treated with curative intent for esophageal and junctional cancer.

## METHODS

### Study Design

This study was a planned secondary analysis of the multicenter ENSURE study (NCT03461341), established by the Young Investigator Division of the European Society of Diseases of the Esophagus.^19^ The ENSURE study was an international multicenter observational cohort study performed across high-volume esophageal cancer centers (n = 20), which aimed among others to determine the impact of intensive surveillance on survival in patients after curative-intent treatment for esophageal or junctional cancer. The primary study outcomes of the ENSURE study have been published previously.^19^ The study is registered on ClinicalTrials.gov (NCT03461341), and with the Research and Innovation Hub, St. James’s Hospital, Dublin, Ireland (approval number #4982) and approved by the St. James’s Hospital and Tallaght University Hospital Joint Research Ethics Committee (approval number #2018-08-CA). Local ethical approvals were obtained in accordance with national policy in participating countries. This planned secondary analysis was approved by the participating centers. To ensure correct structure in the current manuscript, the STROBE guidelines were followed.^20^

### Patient Selection

Patients aged ≥18 years who underwent surgery with curative intent for cTis-4N0-3M0 esophageal or esophagogastric junction (Siewert type I, II, and III) cancer in one of the 20 participating high-volume centers across Europe, between June 2009 and June 2015, were included in the original ENSURE study. Endoscopic therapy or definitive oncological treatment as sole therapy was excluded. Additional exclusion criteria for this current substudy were: other histologic tumor types than adenocarcinoma and squamous cell carcinoma, missing information on surgical approach, salvage surgery (defined as primarily treated with chemoradiation as definitive therapy, but later identified as having locoregional recurrence or residual disease and resected), extended total gastrectomy, Sweet left thoracoabdominal esophagectomy and converted MIE.

### Data Collection and Study Definitions

Data were collected from prospectively maintained databases at the participating centers. Collected data included: sex, age at diagnosis, American Society of Anesthesiologists–grade, Eastern Cooperative Oncology Group performance status, histologic tumor type, clinical tumor and nodal stage, clinical differentiation, tumor site, Siewert type, treatment protocol (surgery only, surgery and adjuvant chemotherapy and/or radiation, neoadjuvant chemotherapy then surgery or neoadjuvant chemoradiation then surgery), operation type (Ivor Lewis, McKeown or transhiatal esophagectomy), robot-assisted surgery, type of conduit, margin status, number of nodes analyzed, number of nodes involved, pathologic tumor and nodal stage, lymphatic/venous invasion and perineural growth, Mandard tumor regression grade, postoperative complications, major morbidity, pulmonary complications, anastomotic leakage, recurrence, type of recurrence and treatment of recurrent disease. The date of recurrence was defined as the date of cytological or histologic confirmation, or of a strong clinical or radiologic suspicion of recurrent disease. Recurrence locations were specified as locoregional (located at the site of the primary tumor or in locoregional lymph nodes), systemic (located systemically or in distant lymph nodes), or combined (coexisting locoregional and systemic recurrences). Treatment for recurrent disease was divided into best supportive care, chemotherapy, chemoradiotherapy, radiotherapy, surgery (with or without radiotherapy, chemotherapy, or chemoradiotherapy), and other local cytoreductive therapies. Disease-free survival (DFS) was defined as the time between esophagectomy and confirmation of recurrence, death, or last follow-up. Overall survival (OS) was defined as the time from surgery to death from any cause or last follow-up. Postrecurrence survival (PRS) was defined as the interval from recurrent disease to death or last follow-up.

### Outcome Measures

Primary endpoints included DFS and recurrence patterns (incidence and location). Secondary endpoints included the operative outcomes; R0 resection rate, lymph node yield, complication rate, pulmonary complication rate, anastomotic leakage rate, and in-hospital mortality. Secondary endpoint also included the oncologic outcomes; treatment of recurrence, OS, and PRS.

### Statistical Analyses

Univariable comparisons were performed using the Student *t* or Mann-Whitney *U* tests for continuous variables, and χ^2^ or Fischer exact test for categorical variables. Data were reported as mean (SD) in case of normal distribution and as median (interquartile range) in case of non-normal distribution. Results of logistic regression analyses were reported with odds ratios (ORs) with 95% CI, results of linear regression with regression coefficient (β) and SE. Results of Cox regression analyses were reported as hazard ratios (HRs) with 95% CI. All multivariable models were adjusted for the following variables: age at diagnosis, sex, American Society of Anesthesiologists–grade, histologic tumor type, clinical T stage, clinical N stage, tumor location, treatment protocol (surgery only, surgery and adjuvant chemotherapy and/or radiation, neoadjuvant chemotherapy then surgery or neoadjuvant chemoradiation then surgery) and operation type (Ivor Lewis, McKeown or transhiatal esophagectomy). All multivariable survival analyses were adjusted for surveillance intensity, since a significant interaction between intensive postoperative surveillance and surgical approach was found (*P* < 0.001). All statistical analyses were conducted by a biostatistician (A.J.) using SAS 9.4 (SAS Institute Inc.). All statistical analyses were 2-tailed, and the threshold of significance was *P* <0.05.

### Sensitivity Analysis

To mitigate the impact of any potential temporal effects with respect to treatment strategy throughout the study (such as increased use of neoadjuvant therapy, reduced use of transhiatal surgery, or changes in anastomotic location), in parallel with increased use of minimally invasive techniques, a sensitivity analysis was performed. All patients who underwent transhiatal esophagectomy were removed from the sensitivity analysis, and it was, in addition, adjusted for time period of surgery (2009–2011 vs 2012–2015).

## RESULTS

### Patient and Tumor Characteristics

The complete ENSURE study cohort included 4793 patients. For this current study, a total of 3199 patients were eligible for inclusion (Supplemental Digital Content Fig. 1, http://links.lww.com/SLA/F57). The study population had a mean age of 64 years (SD: 9.6). The majority of the included patients were males (77.5%). The most common tumor histology was adenocarcinoma (77.5%) and the tumor was most often located in the distal esophagus (47.6%). The majority received neoadjuvant therapy (71.1%; 27.5% chemotherapy and 43.6% chemoradiotherapy). In total, 1748 patients (55%) were operated by OE, 532 (17%) by HMIE, and 919 (29%) by TMIE (Supplemental Digital Content Table 1, http://links.lww.com/SLA/F57).

### Primary Endpoints

#### Disease-free Survival

Median follow-up for the total group was 60.9 months and median DFS 40.3 months (95% CI: 35.6–44.9). Median DFS was 36.5 months (95% CI: 31.0–42.0) for patients who underwent OE, 41.6 months (95% CI: 30.3–528) for patients who underwent HMIE, and 48.7 months (95% CI: 40.1–57.3) for patients who underwent TMIE. Multivariable analysis confirmed that DFS was independently increased among patients post-TMIE [HR: 0.86 (95% CI: 0.76–0.98); *P* = 0.022], but not HMIE [HR: 0.91 (95% CI: 0.77–1.07); *P* = 0.244], as compared with OE (Table 1).

**TABLE 1:** Multivariable Survival Analyses by Surgical Approach

	DFS	Locoregional recurrence-free survival	Distant recurrence-free survival	OS	PRS
	HR (95% CI)	HR (95% CI)	HR (95% CI)	HR (95% CI)	HR (95% CI)
OE	Reference	Reference	Reference	Reference	Reference
HMIE	0.91 (0.77–1.07)	**0.75 (0.56–1.00)**	0.95 (0.77–1.16)	**0.79 (0.67–0.94)**	**0.80 (0.67–0.96)**
TMIE	**0.86 (0.7** **6–0.98)**	0.92 (0.75–1.14)	1.01 (0.86–1.18)	**0.82 (0.72–0.93)**	0.89 (0.77–1.02)

Bold values indicate statistically significant.

Multivariable model was adjusted for the following covariates: age at diagnosis, sex, ASA grade, histologic tumor type, clinical T stage, clinical N stage, tumor location, treatment protocol, operation type, and intensive surveillance.

ASA indicates American Society of Anesthesiologists.

#### Recurrence Patterns

Recurrence was observed among 1423 patients in total (47%), among 799 patients (48.7%) after OE, 233 patients (45.9%) after HMIE, and 391 patients (44.4%) after TMIE (*P* = 0.103). Recurrence was locoregional only in 219 (18.6%), systemic only in 623 (52.8%), and combined locoregional and systemic in 337 (28.6%). On univariable analysis, recurrence pattern was significantly different according to the operative approach (*P* = 0.002). Multivariable logistic regression analysis demonstrated no difference in absolute locoregional recurrence risk according to the operative approach [HMIE vs OE, OR: 0.79 (95% CI: 0.53–1.18), *P* = 0.257; TMIE vs OE, OR: 0.84 (95% CI: 0.62–1.13), *P* = 0.243; Table 2]. However, on multivariable Cox proportional hazards regression, locoregional recurrence-free survival time was increased among patients post-HMIE [HMIE vs OE, HR: 0.75 (95% CI: 0.56–1.00), *P* = 0.046], but not TMIE [TMIE vs OE, HR: 0.92 (95% CI: 0.75–1.14), *P* = 0.455; Table 1].

**TABLE 2:** Multivariable Analysis of Recurrence Patterns by Surgical Approach

	Locoregional recurrence	Systemic recurrence
	OR (95% CI)	OR (95% CI)
OE	Reference	Reference
HMIE	0.79 (0.53–1.18)	**2.07 (1** **.07–4.00** **)**
TMIE	0.84 (0.62–1.13)	0.86 (0.56–1.33)

Bold values indicate statistically significant.

Multivariable model was adjusted for the following covariates: age at diagnosis, sex, ASA grade, histologic tumor type, clinical T stage, clinical N stage, tumor location, treatment protocol, and operation type.

ASA indicates American Society of Anesthesiologists.

The probability of systemic recurrence as the site of first treatment failure was independently increased among patients post-HMIE [HMIE vs OE, OR: 2.07 (95% CI: 1.07–4.00), *P* = 0.031], but not TMIE [TMIE vs OE, OR: 0.86 (95% CI: 0.56–1.33), *P* = 0.508], although time to distant recurrence was equivalent according to approach [HMIE vs OE, HR: 0.95 (95% CI: 0.77–1.16), *P* = 0.582; TMIE vs OE, HR: 1.01 (95% CI: 0.86–1.18), *P* = 0.954; Table 1].

### Secondary Endpoints

#### Operative Outcomes

Most of the patients had a radical surgical resection without positive tumor margins (88.0% R0), the mean lymph node yield was 24.3 (SD: 11.2). When specified for surgical approach, R0 resection was more often achieved after TMIE (93.2%), as compared with HMIE (86.9%) and OE (85.6%; *P* < 0.001; Supplemental Digital Content Table 1, http://links.lww.com/SLA/F57). On multivariable analysis, the probability of R0 resection was independently increased following TMIE [HMIE vs OE, OR: 0.86 (95% CI: 0.59–1.25), *P* = 0.424; TMIE vs OE, OR: 0.62 (95% CI: 0.45–0.86), *P* = 0.005]. A higher mean number of lymph nodes was retrieved in patients after TMIE (26.8) as compared with HMIE (24.7) and OE (22.9, *P* < 0.001, Supplemental Digital Content Table 1, http://links.lww.com/SLA/F57). Multivariable linear regression confirmed independently improved nodal yield among patients undergoing TMIE (β = 3.18, SE: 0.47, *P* < 0.001), but not HMIE (β = 0.41, SE: 0.62 *P* = 0.512).

Complication data were available for 3152 patients (98.5%). There was no difference in the incidence of any complication, or severe complications according to surgical approach (*P* = 0.101 and *P* = 0.554, respectively, Supplemental Digital Content Table 1, http://links.lww.com/SLA/F57). Multivariable analysis confirmed that there was no difference in the incidence of overall complications [HMIE vs OE, OR: 0.81 (95% CI: 0.64–1.01), *P* = 0.100; TMIE vs OE, OR: 0.86 (95% CI: 0.71–1.03), *P* = 0.107], or major complications [HMIE vs OE, OR: 0.84 (95% CI: 0.63–1.12), *P* = 0.235; TMIE vs OE, OR: 0.91 (95% CI: 0.73–1.13), *P* = 0.405] between groups (Table 3).

**TABLE 3:** Multivariable Analysis of Complications by Surgical Approach

	Any complication	Major complication	Pulmonary complication	Anastomotic leakage
	OR (95% CI)	OR (95% CI)	OR (95% CI)	OR (95% CI)
OE	Reference	Reference	Reference	Reference
HMIE	0.81 (0.64–1.01)	0.84 (0.63–1.12)	0.79 (0.62–1.01)	1.14 (0.79–1.64)
TMIE	0.86 (0.71–1.03)	0.91 (0.73–1.13)	**0.78 (0.64** **–** **0.94)**	**1.71 (1.34** **–** **2.20)**

Bold values indicate statistically significant.

Multivariable model was adjusted for the following covariates: age at diagnosis, sex, ASA grade, histologic tumor type, clinical T stage, clinical N stage, tumor location, treatment protocol, and operation type.

ASA indicates American Society of Anesthesiologists.

Pulmonary complications occurred less frequently among patients undergoing TMIE, but not HMIE, as compared with OE, on univariable (OE: 39.6%, HMIE: 33.3%, and TMIE: 34.7%; *P* = 0.006, Supplemental Digital Content Table 1, http://links.lww.com/SLA/F57) and multivariable analysis [HMIE vs OE, OR: 0.79 (95% CI: 0.62–1.01), *P* = 0.062; TMIE vs OE, OR: 0.78 (95% CI: 0.64–0.94), *P* = 0.008, Table 3]. Anastomotic leakage was more often observed after TMIE (18.2%) as compared with after HMIE (10.9%) and OE (12.3%; *P* < 0.001, Supplemental Digital Content Table 1, http://links.lww.com/SLA/F57), and was independently increased among patients undergoing TMIE on multivariable analysis as well [HMIE vs OE, OR: 1.14 (95% CI: 0.79–1.64), *P* = 0.480; TMIE vs OE, OR: 1.71 (95% CI: 1.34–2.20), *P* < 0.001; Table 3). The risk of in-hospital mortality was equivalent between approaches (*P* = 0.437).

#### Oncologic Outcomes

Treatment of recurrence was known for all patients with recurrence (n = 1423); of whom 46.3% (n = 659) received best supportive care, and the remaining 53.7% (n = 764) of the patients received tumor-directed therapy. Radiotherapy was used in 398 patients, 543 received chemotherapy, 110 patients had surgical resection and 8 patients underwent other local cytoreductive therapies. When specifying the treatment by surgical approach, tumor-directed therapy was more frequently administered after HMIE (56.7%), as compared with OE (55.6%) or TMIE (48.1%; *P* = 0.038). However, on multivariable analysis, including adjustment for intensive surveillance [OR: 2.24 [95% CI: 1.64–3.05), *P* < 0.001], no difference in the probability of tumor-directed therapy was observed according to the operative approach [HMIE vs OE, OR: 1.31 (95% CI: 0.89–1.93), *P* = 0.166; TMIE vs OE, OR: 0.86 (95% CI: 0.64–1.15), *P* = 0.314; Table 4).

**TABLE 4:** Multivariable Analysis of Treatment of Recurrence by Surgical Approach

	Tumor-directed therapy
	OR (95% CI)
OE	Reference
HMIE	1.31 (0.89–1.93)
TMIE	0.86 (0.64–1.15)

Multivariable model was adjusted for the following covariates: age at diagnosis, sex, ASA grade, histologic tumor type, clinical T stage, clinical N stage, tumor location, treatment protocol, and operation type.

ASA indicates American Society of Anesthesiologists.

**FIGURE 1:**
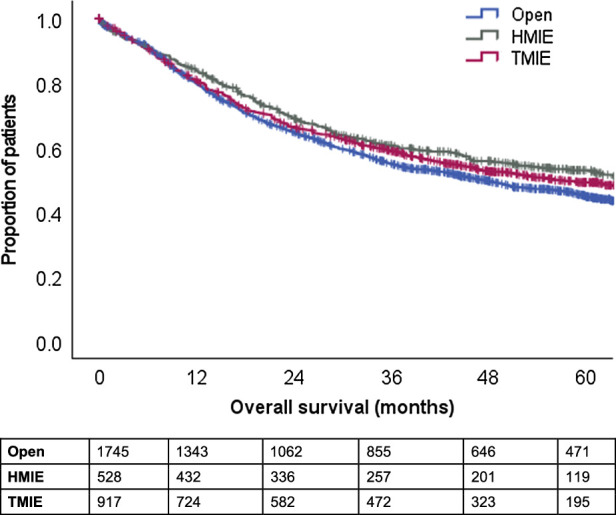
OS curves, specified for surgical approach; *P* = 0.024.

OS data were available for 3275 patients with a median OS of 51.5 months (95% CI: 46.9–56.1). When specified for the surgical approach, median OS was 47.1 months (95% CI: 41.3–52.9) after OE, 63.9 months (95% CI: 50.3–77.6) after HMIE and 56.1 months (interquartile range: 47.5–64.7) after TMIE (*P* = 0.024, Fig. 1). Multivariable analysis showed that OS was independently improved after both HMIE [HR: 0.79 (95% CI: 0.67–0.94), *P* = 0.009] and TMIE [HR: 0.82 (95% CI: 0.72–0.93), *P* = 0.003) as compared with OE in this study (Table 1).

**FIGURE 2:**
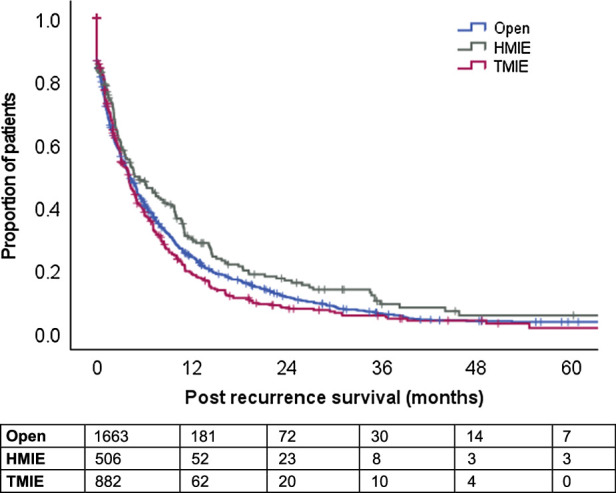
PRS curves, specified for surgical approach; *P* = 0.047.

Median PRS was 4.1 months (95% CI: 3.6–4.6 mo). When specified for the surgical approach, PRS was superior for patients underwent HMIE [4.8 mo (95% CI: 2.7–6.8) vs 4.1 mo (95% CI: 3.4–4.7) in case of OE and 4.0 mo (95% CI: 3.4–4.7) for TMIE, *P* = 0.047; Fig. 2]. Multivariable analysis adjusting for surveillance intensity confirmed greater PRS among patients post-HMIE [HR: 0.80 (95% CI: 0.67–0.96), *P* = 0.017] but not post-TMIE [HR: 0.89 (95% CI: 0.77–1.02), *P* = 0.084] as compared with OE (Table 1).

### Sensitivity Analysis

In the sensitivity analysis, results with regard to both recurrence patterns and survival remained significantly better for minimally invasive surgery, with no change in the overall findings of the study. Results of sensitivity analyses are shown in Supplemental Tables 2 (Supplemental Digital Content 1, http://links.lww.com/SLA/F57) and 3 (Supplemental Digital Content 1, http://links.lww.com/SLA/F57).

## DISCUSSION

This large multicenter observational cohort study including prospectively collected data from 20 high-volume centers across Europe and North America investigated operative and oncologic outcomes after esophagectomy for esophageal or esophagogastric junction cancer performed by either an open, hybrid, or total minimally invasive approach. OS was independently improved after both HMIE and TMIE, as compared with OE. This manifested through prolonged DFS post-TMIE and improved PRS post-HMIE.

After TMIE, a higher rate of R0 resections, a higher lymph node yield, and fewer pulmonary complications were observed. This superiority on several surgical quality indicators, probably as a result of better visualization and more precise lymph node dissection during minimally invasive surgery, might have led to more optimal local tumor control, reducing recurrence rate and prolonging survival. As a result of extensive lymphadenectomy, the clearance of malignant lymphatic tissue is maximized, improving DFS, and hence OS.^21,22^ This is strengthened by the results of a randomized controlled trial suggesting a possible improvement in oncologic outcomes after minimally invasive, as compared with open, esophagectomy.^23^ In the traditionally invasive vs. minimally invasive esophagectomy trial, although not statistically significant, 3-year DFS was 40.2% after TMIE as compared with 35.9% after OE, whereas 3-year OS of 50.5% after TMIE compared with 40.4% after OE was observed. In the current study, although the operative approach was not found to be independently predictive of overall recurrence rate, after TMIE, the time to disease recurrence was prolonged compared with OE.

After HMIE, a prolonged locoregional recurrence-free interval was observed, potentially also indicating relative enhancements in surgical quality and local disease control within this cohort of patients (as reflected by higher lymph node yield and fewer pulmonary complications than after OE in univariable analyses). It appears that rather than experiencing locoregional failure, patients post-HMIE are more prone to systemic failure, as evidenced by the higher risk of distant recurrence observed among patients post-HMIE. Another explanation for improved OS after HMIE is probably the reduced surgical trauma associated with the procedure. It is notable that patients undergoing HMIE experienced equivalent reductions in pulmonary complications as TMIE, but with leakage rates comparable to open surgery. This balance between minimization of pulmonary complications and faster postoperative recovery, without increased risk of anastomotic leakage seen in the TMIE group, may result in better preservation of preoperative performance status among patients undergoing HMIE, as compared with after open surgery. However, it is also possible that unmeasured confounding may exist between groups due to, for example, case selection during the initial transition from open to minimally invasive approaches and proficiency gain curve effect in the MIE groups.

Of patients, 47% in this European esophageal cancer patient cohort developed recurrent disease during follow-up, which is commensurate with the 45% in the recently published IVORY study from the Netherlands, describing a Dutch nationwide cohort of patients with esophageal cancer treated with either neoadjuvant chemotherapy or chemoradiotherapy and surgery.^16^ Comparable results were also described in another Dutch two-institutional retrospective cohort study investigating the recurrence patterns after CROSS chemoradiotherapy and surgery with curative intent, in which a recurrence incidence of 45% was found. The current European results demonstrating improved DFS after TMIE as compared with OE support the association between OE and recurrence that was observed in the IVORY study.^16^

This large European study of 3199 patients provides an extensive overview of operative and oncologic outcomes for patients with esophageal cancer after both OE and MIE. Because of its large and heterogeneous population, the results are generalizable to the European esophageal cancer patient cohort that undergoes an esophagectomy in a high-volume center. A number of limitations are acknowledged. The evolution of evidence during the time frame of this study may have led to variation in initial treatment, surgical techniques, as well as treatment of recurrence, occurring in concert with the implementation of minimally invasive techniques. The OE group in the present study included patients undergoing both transthoracic (n = 1418, 81.1%) and transhiatal (n = 330, 18.9%) resection. As transhiatal surgery may be associated with reduced lymph node clearance in the chest, this may have biased the OE group towards increased locoregional recurrence. However, to limit the impact of this factor, and to mitigate the effect of time lead bias, all multivariable models were adjusted for treatment protocol [eg, whether or not (neo)adjuvant therapy was used] and operation type (transthoracic Ivor Lewis, transthoracic McKeown or transhiatal). Furthermore, sensitivity analyses were undertaken which excluded patients who underwent transhiatal resection and were adjusted for the time period within the study (2012–2015 vs 2009–2011). Sensitivity analyses (Supplemental Digital Content Table 2, http://links.lww.com/SLA/F57 and Supplemental Digital Content Tables 3, http://links.lww.com/SLA/F57) showed no difference in findings, confirming the robustness of the conclusions described herein.

## CONCLUSIONS

In this European multicenter study, clear differences in recurrence patterns, survival, and operative outcomes were observed according to the esophagectomy approach. HMIE was associated with improved local recurrence-free survival time, and increased probability of distant recurrence as the first site of treatment failure, with improved PRS on multivariable analysis, as compared with OE. In contrast, TMIE was associated with an independent improvement in DFS as compared with OE. Both HMIE and TMIE were associated with improved OS compared with OE, which may have been mediated by enhanced surgical quality indicators and therewith improved tumor control after minimally invasive surgery. Optimum oncologic outcomes may be achieved after TMIE; however, this was at the expense of increased anastomotic leakage rates in the present series, representing the initial adoption of TMIE in Europe. The current findings suggest that critical factors such as unit experience and training, as well as patient-reported outcome measures, should guide decision-making toward the most suitable operative approach for patients with esophageal cancer.

## Supplementary Material
