# Supplementary material for: Recurrence and Survival After Minimally Invasive and Open Esophagectomy for Esophageal Cancer: A Post Hoc Analysis of the Ensure Study
Source: Ann Surg. 2024 Apr 5;280(2):267–73. doi: 10.1097/SLA.0000000000006280 (PMC11224562; doi:10.1097/SLA.0000000000006280)
Supplement: Supplementary file 1 [file sla-280-267-s001.docx]

**SUPPLEMENTARY DATA FILE**


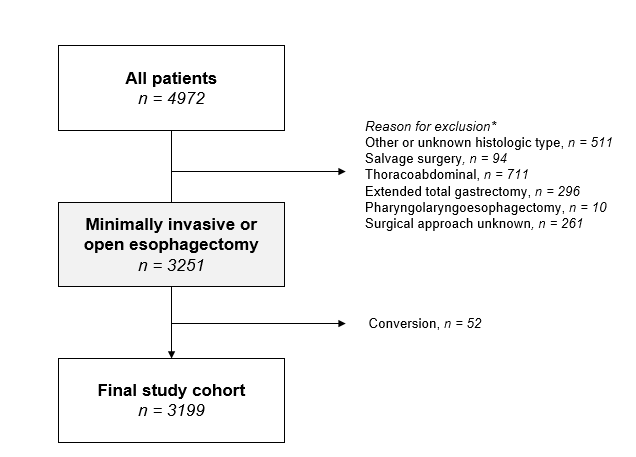


**Supplementary Figure 1.** Flowchart of patient selection

*patients may meet more than one exclusion criterion

| **Supplmentary Table 1.** Clinicopathologic and treatment characteristics oncological and postoperative outcomes, stratified by surgical approach | | | | |
| --- | --- | --- | --- | --- |
|  | **All patients**  **n = 3,199** | **OE**  **n = 1,748** | **HMIE**  **n = 532** | **TMIE**  **n = 919** |
| **Clinicopathologic characteristics** | | | | |
| **Sex, n (%)**  Male  Female | 2478 (77.5)  721 (22.5) | 1323 (75.7)  425 (24.3) | 447 (84.0)  85 (16.0) | 708 (77.0)  211 (23.0) |
| **Age at diagnosis, mean (SD)** | 64 (9.4) | 64.3 (9.6) | 64.1 (9.9) | 63.6 (9.5) |
| **ASA-grade, n (%)**  Grade I  Grade II  Grade III | 860 (27.3)  1599 (50.8)  690 (21.9) | 512 (29.5)  854 (49.3)  367 (21.2) | 149 (29.7)  258 (51.5)  94 (18.8) | 199 (21.7)  487 (53.2)  229 (25.0) |
| **ECOG performance status, n (%)**  0  1  2  3 | 1374 (62.4)  720 (32.7)  93 (4.2)  14 (0.6) | 831 (62.9)  422 (31.9)  57 (4.3)  12 (0.9) | 239 (59.8)  138 (34.5)  21 (5.3)  2 (0.5) | 304 (63.5)  160 (33.4)  15 (3.1)  0 |
| **Histologic type, n (%)**  Adenocarcinoma  Squamous cell carcinoma | 2478 (77.5)  721 (22.5) | 1319 (75.5)  429 (24.5) | 442 (83.1)  90 (16.9) | 717 (78.0)  202 (22.0) |
| **Clinical T stage, n (%)**  cTis/HGD  cT1  cT2  cT3  cT4 | 40 (1.3)  372 (12.5)  592 (19.9)  1863 (62.7)  105 (3.5) | 19 (1.2)  195 (12.0)  319 (19.6)  1028 (63.3)  63 (3.9) | 10 (2.1)  68 (14.6)  96 (20.6)  282 (60.5)  10 (2.1) | 11 (1.2)  109 (12.4)  177 (20.1)  553 (62.7)  32 (3.6) |
| **Clinical N stage, n (%)**  cN0  cN1  cN2  cN3 | 1152 (38.6)  1277 (42.8)  503 (16.9)  49 (1.6) | 632 (38.9)  724 (44.6)  244 (15.0)  23 (1.4) | 159 (34.1)  182 (39.1)  119 (25.5)  6 (1.3) | 361 (40.5)  371 (41.6)  140 (15.7)  20 (2.2) |
| **Clinical differentiation, n (%)**  Cannot be assessed  Well differentiated  Moderately differentiated  Poorly differentiated  Signet ring | 381 (19.3)  130 (6.6)  729 (36.9)  570 (28.8)  167 (8.4) | 147 (12.7)  74 (6.4)  460 (39.7)  380 (32.8)  98 (8.5) | 97 (28.2)  40 (11.6)  124 (36.0)  68 (19.8)  15 (4.4) | 137 (28.9)  16 (3.4)  145 (30.6)  122 (25.7)  54 (11.4) |
| **Tumor site, n (%)**  Junctional  Lower  Middle  Upper | 1190 (37.9)  1494 (47.6)  420 (13.4)  35 (1.1) | 773 (44.9)  660 (38.3)  271 (15.7)  19 (1.1) | 205 (40.4)  259 (51.0)  40 (7.9)  4 (0.8) | 212 (23.3)  575 (63.3)  109 (12.0)  12 (1.3) |
| **Siewert type, n (%)**  AEG 1  AEG 2  AEG 3 | 813 (54.1)  614 (40.8)  75 (5.0) | 444 (50.7)  382 (43.6)  50 (5.7) | 112 (59.3)  71 (37.6)  6 (3.2) | 257 (58.8)  161 (36.8)  19 (4.3) |
| **Treatment characteristics** | | | | |
| **Treatment protocol**  Surgery only  Surgery and adjuvant chemo(radio)therapy  Chemotherapy + surgery  Chemoradiotherapy + surgery | 821 (25.7)  102 (3.2)  881 (27.5)  1395 (43.6) | 454 (26.0)  85 (4.9)  527 (30.1)  682 (39.0) | 146 (27.4)  11 (2.1)  206 (38.7)  169 (31.8) | 221 (24.0)  6 (0.7)  148 (16.1)  544 (59.2) |
| **Operation type**  Ivor Lewis  McKeown  Transhiatal | 1745 (54.5)  1040 (32.5)  414 (12.9) | 942 (53.9)  476 (27.2)  334 (19.1) | 420 (78.9)  108 (20.3)  0 | 383 (41.7)  456 (49.6)  80 (8.7) |
| **Robot-assisted surgery**  No  Yes | 3056 (95.5)  143 (4.5) | 1748 (100)  0 | 532 (100)  0 | 776 (84.4)  143 (15.6) |
| **Oncological outcomes** | | | | |
| **Margin status, n (%)**  R0  R1  R2 | 2775 (88.0)  371 (11.8)  8 (0.3) | 1486 (85.6)  244 (14.1)  6 (0.3) | 444 (86.9)  67 (13.1)  0 | 845 (93.2)  60 (6.6)  2 (0.2) |
| **Number of nodes analyzed, mean (SD)** | 24.3 (11.2) | 22.9 (10.6) | 24.7 (11.1) | 26.8 (11.7) |
| **Number of nodes involved, mean (SD)** | 1.94 (4.0) | 2.06 (3.9) | 2.02 (4.0) | 1.68 (4.1) |
| **Pathologic T stage**  T0  Tis/HGD  T1  T2  T3  T4 | 472 (14.8)  51 (1.6)  713 (22.4)  451 (14.1)  1399 (43.9)  104 (3.3) | 228 (13.1)  32 (1.8)  347 (19.9)  250 (14.3)  816 (46.8)  71 (4.1) | 64 (12.1)  8 (1.5)  131 (24.7)  80 (15.1)  237 (44.6)  11 (2.1) | 180 (19.7)  11 (1.2)  235 (25.7)  121 (13.2)  346 (37.8)  22 (2.4) |
| **Pathologic N stage**  N0  N1  N2  N3 | 1750 (54.8)  659 (20.6)  543 (17.0)  242 (7.6) | 924 (53.0)  369 (21.1)  304 (17.4)  148 (8.5) | 273 (51.4)  105 (19.8)  119 (22.4)  34 (6.4) | 553 (60.2)  185 (20.2)  120 (13.1)  60 (6.5) |
| **Lymphatic invasion**  Not present  Present | 1627 (71.5)  650 (28.5) | 872 (68.8)  396 (31.2) | 242 (68.2)  113 (31.8) | 513 (78.4)  141 (21.6) |
| **Venous invasion**  Not present  Present | 1844 (73.1)  680 (26.9) | 970 (71.8)  381 (28.2) | 294 (76.8)  89 (23.2) | 580 (73.4)  210 (26.6) |
| **Perineural invasion**  Not present  Present | 1826 (78.0)  515 (22.0) | 962 (76.0)  304 (24.0) | 282 (76.4)  87 (23.8) | 582 (82.4)  124 (17.6) |
| **Mandard tumor regression grade**  Not applicable  TRG 1  TRG 2  TRG 3  TRG 4  TRG 5 | 1267 (39.6)  441 (13.8)  372 (11.6)  428 (13.4)  462 (14.4)  229 (7.2) | 732 (41.9)  211 (12.1)  187 (10.7)  221 (12.6)  269 (15.4)  128 (7.3) | 235 (44.2)  58 (10.9)  57 (10.7)  50 (9.4)  85 (16.0)  47 (8.8) | 300 (32.6)  172 (18.7)  128 (13.9)  157 (17.1)  108 (11.8)  54 (5.9) |
| **Postoperative outcomes** | | | | |
| **Postoperative complications**  No  Yes | 1207 (38.3)  1945 (61.7) | 637 (36.8)  1094 (63.2) | 214 (41.8)  298 (58.2) | 356 (39.2)  553 (60.8) |
| **Major morbidity (Clavien-Dindo ≥3b)**  No  Yes | 2446 (78.4)  675 (21.6) | 1328 (77.8)  379 (22.2) | 409 (80.0)  102 (20.0) | 709 (78.5)  194 (21.5) |
| **Anastomotic leakage**  No leakage  Leakage | 2719 (86.2)  435 (13.8) | 1522 (87.7)  214 (12.3) | 456 (89.1)  56 (10.9) | 741 (81.8)  165 (18.2) |
| **Pulmonary complications**  No pulmonary complications  Pulmonary complications | 1790 (63.0)  1050 (37.0) | 920 (60.4)  604 (39.6) | 275 (66.7)  137 (33.3) | 595 (65.8)  309 (34.2) |
| **In-hospital mortality**  No  Yes | 3032 (96.0)  126 (4.0) | 1662 (95.7)  75 (4.3) | 493 (96.3)  19 (3.7) | 877 (96.5)  32 (3.5) |

*Abbreviations*: OE = Open Esophagectomy, HMIE = Hybrid Minimally Invasive Esophagectomy, TMIE = Total Minimally Invasive Esophagectomy, ECOG = Eastern Cooperative Oncology Group, ASA = American Society of Anesthesiologists, AEG = Adenocarcinoma of the Esophago Gastric junction, TRG = Tumor Regression Grade

**Supplementary Table 2.** Sensitivity multivariable analysis of recurrence patterns among patients undergoing transthoracic esophagectomy.

| **SENSITIVITY ANALYSIS** | | |
| --- | --- | --- |
|  | **Locoregional recurrence** | **Systemic recurrence** |
|  | **OR (95% CI)** | **OR (95% CI)** |
| *OE* | *Reference* | *Reference* |
| HMIE | 0.75 (0.50-1.14) | **1.98 (1.01-3.88)** |
| TMIE | 0.76 (0.55-1.06) | 0.85 (0.53-1.36) |
| Multivariable model was adjusted for the following covariates: age at diagnosis, sex, ASA-grade, histologic tumor type, clinical T stage, clinical N stage, tumor location, treatment protocol, operation type (Ivor Lewis vs. McKeown) and year of surgery.  Statistically significant values are highlighted in bold.  *Abbreviations*: OE = Open Esophagectomy, HMIE = Hybrid Minimally Invasive Esophagectomy, TMIE = Total Minimally Invasive Esophagectomy, OR = Odds Ratio | | |

**Supplementary Table 3.** Sensitivity multivariable analysis of recurrence and survival among patients undergoing transthoracic esophagectomy.

| **SENSITIVITY ANALYSIS** | | | | | |
| --- | --- | --- | --- | --- | --- |
|  | **Disease-free survival** | **Locoregional recurrence-free survival** | **Distant recurrence-free survival** | **Overall survival** | **Post-recurrence survival** |
|  | **HR (95% CI)** | **HR (95% CI)** | **HR (95% CI)** | **HR (95% CI)** | **HR (95% CI)** |
| *OE* | *Reference* | *Reference* | *Reference* | *Reference* | *Reference* |
| HMIE | 0.90 (0.77-1.07) | **0.73 (0.55-0.98)** | 0.95 (0.77-1.16) | **0.80 (0.67-0.95)** | **0.80 (0.66-0.96)** |
| TMIE | **0.82 (0.72-0.95)** | 0.89 (0.70-1.13) | 1.02 (0.86-1.21) | **0.79 (0.69-0.91)** | 0.84 (0.73-0.98) |
| Multivariable model was adjusted for the following covariates: age at diagnosis, sex, ASA-grade, histologic tumor type, clinical T stage, clinical N stage, tumor location, treatment protocol, operation type (Ivor Lewis vs. McKeown), intensive surveillance and year of surgery.  Statistically significant values are highlighted in bold.  *Abbreviations*: OE = Open Esophagectomy, HMIE = Hybrid Minimally Invasive Esophagectomy, TMIE = Total Minimally Invasive Esophagectomy, HR = Hazard Ratio | | | | | |
